# Industrial Waste Treatment by ETS-10 Ion Exchanger Material

**DOI:** 10.3390/ma11112316

**Published:** 2018-11-18

**Authors:** Pierantonio De Luca, Ivano Bernaudo, Rosangela Elliani, Antonio Tagarelli, Jànos B. Nagy, Anastasia Macario

**Affiliations:** 1 Dipartimento di Ingegneria Meccanica, Energetica e Gestionale, Università della Calabria, Arcavacata di Rende, 87036 Rende (Cs), Italy; 2 Dipartimento di Ingegneria per l’Ambiente ed il Territorio ed Ingegneria Chimica, Università della Calabria, Arcavacata di Rende, 87036 Rende (Cs), Italy; bernaudo.ivano@gmail.com (I.B.); janos.bnagy1@gmail.com (J.B.N.); 3 Dipartimento di Chimica e Tecnologie Chimiche, Università della Calabria, Arcavacata di Rende, 87036 Rende (Cs), Italy; rosangela.elliani@hotmail.it (R.E.); antonio.tagarelli@unical.it (A.T.)

**Keywords:** ETS-10, industrial waste, zinc ferrite, ionic exchange, titanium silicate

## Abstract

The aim of this project was to study the treatment of industrial waste using ETS-10 zeolite. The pollutants that must be removed were metals sourced from zinc ferrite, a processing waste derived from the use of mineral-containing zinc. The first phase of the work involved the characterization of the industrial waste, zinc ferrite, in order to deepen the knowledge regarding its nature and composition. The second phase involved the removal of the metals released by the zinc ferrite in aqueous systems using the ETS-10 phase as an ion exchanger. Different chemical and physical techniques were used: plasma mass spectrometry, X-ray diffraction, scanning electron microscopy, microanalysis, and thermal analyses. A comparison between ETS-10 and commercial zeolite A performance, in the same aqueous systems, was carried out. The results showed that the metal removal efficiency of ETS-10 phase is higher than that obtained by commercial zeolite A, especially towards dangerous heavy metals such as Pb, Zn and Mn.

## 1. Introduction

Industrial waste may contain a large quantity of pollutants and the composition depends on the type of production process. The impact of these pollutants on the environment is considerable and complex, since the amounts of different harmful substances contained in these wastes increase their toxicity [1]. Heavy metals are often found in industrial waste water and, even though they are initially present in low concentrations, the heavy metal content may increase over time because they are not biodegradable [2]. The alteration of soil by heavy metal presence may affect aquatic and terrestrial ecosystems.

In order to reduce the levels of both organic and inorganic heavy metals and pollutants, different materials are used, each with particular characteristics, such as ion exchange capacity, adsorption, and photocatalytic action. Zeolites [3,4,5,6,7,8], geopolymers [9,10,11], activated carbons [12], nanomaterials [13,14], and plants with a phytodepurative action [15] are some examples of these materials.

Generally, the ion exchange process is widely used for the removal of heavy metals in the treatment of waste water due to its high treatment capacity, high removal efficiency, and fast kinetics [16]. Many researchers have demonstrated that zeolites have an excellent exchange capacity for metal removal, even under different operating conditions [17,18,19]. The main disadvantage of this process is that the exchanger can be easily contaminated by any organic substances that strongly reduces its exchange capacity [20,21].

In this work, we report the synthesis and application of ion exchanger material for zinc ferrite removal. The material proposed is ETS-10, largely used as efficient ion exchanger [22]. The main advantages of the use of ETS-10 as an ion exchanger are its easy regenerability and its high thermal stability. ETS is stable up to a temperature of 550 °C [23]. This means that when it is polluted by organic substances, it can be regenerated by calcination. Finally, ETS is stable under very alkaline pH conditions. Although there is no real market for this material yet, its use is economical because its synthesis procedure is simple: by hydrothermal reaction and using very cheap raw materials [24].

The synthesized ion exchanger belongs to the Engelhard Titanium Silicate (ETS) family. There are several varieties of ETS materials, but the most common are the ETS-10 and ETS-4 phases. Both these phases were synthesized for the first time by Kuznicki [24,25]. The ETS-10 phase is an extremely interesting titanosilicate microporous material due to its high thermal stability and wide pores (pore size close to 0.8 nm) [26,27,28,29,30]. Many studies focused on the synthesis of these materials, as well as the study of their structure [31,32,33,34,35,36,37,38]. Further studies examined the possibility of inserting hetero-atoms into the structure of these materials in order to improve or modify their characteristics [39,40,41]. These materials are versatile and can be applied in a variety of fields, such as water purification and heavy metal removal [42,43,44,45,46,47], gas adsorption [48,49,50], and photo-catalysis [51,52]. Generally, after synthesis, these materials are obtained in the form of powders.

Considerable research has addressed the synthesis of microporous materials in the pellet form for different applications [53,54,55]. Pellet preparation, involving the use of a binder, may obstruct the porous structure of the main material, resulting in decreased accessibility to its pores. This procedure strong reduces the ion exchange, molecular sieving, adsorption, and catalysis capacity of the final material in pellet form. As such, some research has focused on the preparation of pellets of microporous and zeolite materials without the use of a binder [56,57,58]. In order to obtain self-bonded pellets by direct synthesis, the conditions were studied for ETS without the use of a binder [59,60]. Further research included heteroatoms inside pellets with the aim of modifying their characteristics [61,62]. Studies have reported self-bonded ETS-10 and ETS-4 pellets with carbon nanotube insertions [63].

Zinc ferrite is an industrial waste derived from the thermal treatment of zinc extraction from blenda, a mineral including zinc sulfide. This waste, which appears as dust, is generally stored in open landfills where it is easily exposed to rainwater or surface waters that can promote the release of heavy metals contained within, potentially damaging the environment and human health [64].

The aim of the present research was dual: a full characterization of the waste (zinc ferrite) in order to increase our knowledge on the nature and the chemical composition of zinc ferrite, and to test the capacity of the ETS-10 ion exchanger to remove metals from water contaminated by zinc ferrite. To the best of our knowledge, there are no studies on the treatment of water contaminated by zinc ferrite using the ETS-10 material as ion exchanger. For this reason, our work could be the pioneer in this area. Finally, we compared the metal removal capacity of ETS-10 with that of a commercial zeolite A, in the same aqueous systems contaminated by zinc ferrite. Zeolite A is a material largely used in different applications, as a filler, adsorbent or catalyst [65,66,67], but its main use is as an ion exchanger [68,69,70].

## 2. Materials and Methods

### 2.1. Materials

The main materials studied and used in this research included industrial waste, zinc ferrite, and the ETS-10 phase. The utilized zinc ferrite was obtained from an Italian metallurgical industry assigned to the production of zinc. The ETS-10 phase was used as an ionic exchanger for the removal of the metals released by the zinc ferrite in aqueous solutions. The used ETS-10 phase was in sodium-potassium form and can produce ion-exchange reactions, as shown in the following simplified expression: (Na, K) ETS-10 + M^+2^ ⇄ (M) ETS-10 + Na^+^ + K^+^. ETS-10 was synthesized according to the procedures of De Luca et al. [48] and by using the following system for the initial gel preparation: 1.0Na_2_O-0.6KF-0.2TiO_2_-1.28HCl-1.49SiO_2_-39.5H_2_O. Synthesis consisting of two different batches, an acidic batch made of HCl (37 wt %, Carlo Erba, Milan, Italy), TiCl_4_ (50 wt %, Carlo Erba, Milan, Italy), KF (40 wt %, Merck, Darmstadt, Germany), and distilled water; and a basic batch made of sodium silicate (8 wt % of Na_2_O, 27 wt % SiO_2_, Merck, Darmstadt, Germany) and NaOH (50 wt %, BDH Anala R, Milan, Italy). Once mixed together, the two solutions were stirred for several minutes. Subsequently, the gel was introduced into Morey-type autoclaves and were placed into an oven at 190 °C for a reaction time of 48 h. Commercial zeolite A was supplied by Sigma-Aldrich, Darmstadt, Germany).

### 2.2. Characterization

Characterization was carried out by the use of different instruments and analyses: X-ray diffractometry (XRD-APD 2000 PRO, GNR, Novara, Italy), thermal analyses (TGA and DTA-DTG-60, Shimadzu, Kyoto, Japan), scanning electron microscope (SEM; PHENOM Prox, Eindhoven, The Netherlands), and microanalysis (EDS; PHENOM ProX, Eindhoven, The Netherlands), conductivity meter, and pH meter (VWR phenomenal 1100L, Milan, Italy). Inductively coupled plasma mass spectrometry was performed using an ICP-MS (Perkin-Elmer Elan DRC, Seattle, WA, USA). For this analysis, several isotopes were used for the same element in order to evaluate the greater reliability between different isotopes monitored: ^27^Al, ^138^Ba, ^9^Be, ^209^Bi, ^44^Ca, ^114^Cd, ^59^Co, ^52^Cr, ^53^Cr, ^133^Cs, ^63^Cu, ^56^Fe, ^57^Fe, ^54^Fe, ^69^Ga, ^115^In, ^39^K, ^7^Li, ^24^Mg, ^55^Mn, ^23^Na, ^58^Ni, ^208^Pb, ^85^Rb, ^88^Sr, ^205^Tl, ^238^U, ^64^Zn, and ^66^Zn. The experimental conditions were: plasma power 1100 W, gas flow rate in the nebulizer 0.75 mL/min, quartz nebulizer, CeO/Ce oxide ratio <3%, sample flow 1 mL/min, argon refrigerant flow speed 15 L/m, dwell time 50 ms, scan mode Peak hopping, sweeps/reading 40, reading/replicate 1, and 3 replicates.

### 2.3. Preparation of the Samples and Aqueous Systems

In order to measure pH and conductivity, 25 g of ferrite were dispersed in 200 mL distilled water, which was then subjected to magnetic stirring at room temperature.

The zinc ferrite sample was treated at 100 °C for 2 h to remove water moisture before proceeding with the experimentation, which was subsequently homogenized by a mortar mill. Part of the prepared sample underwent a mineralization process and digestion with acidic attack, in order to determine the concentration of metallic species characterizing zinc ferrite by mass spectrometry with ICP-MS plasma [71]. Nitric acid, concentrated at 65%, and hydrogen peroxide, concentrated at 30%, were used as reagents. Initially, 0.5 g of calcined zinc ferrite at 200 °C and 10 mL of HNO_3_ were placed inside a beaker. The prepared system was left at room temperature for 2 h.

Subsequently, the suspension was placed on a heated plate to digest and stirred for 2 h, followed by a cooling time of 30–40 min. Following this, the required volume of HNO_3_ and H_2_O_2_ was added in order to obtain a clear solution. This solution was then brought to a volume with 100 mL using distilled H_2_O. By doing so, all the zinc ferrite was completely dissolved into solution, which contained all the elements that compose zinc ferrite, in preparation for ICP-MS analysis. 

The zinc ferrite, once characterized, determined the concentration and the nature of the elements present therein. Then, we moved to the second phase of the work. 

### 2.4. Ion Exchanger Procedures

The last phase involved the use of the ion exchanger for the removal of the elements released by the zinc ferrite in aqueous systems. With this object in mind, we prepared the following systems: four systems were obtained by mixing 5 g of ferrite in 100 mL of distilled water (blanks, samples A), another four systems were obtained by adding to the blanks 10 g of ETS-10 (samples B) and the last four were obtained by adding to the blanks 10 g of zeolite A (samples C). Samples with ion exchanger were stirred at room temperature for the following times: 0.5, 1.0, 1.5, and 2.0 h (see Table 1). The ion exchanger was immersed into the systems in a closed sachet of filter paper in order to facilitate its final recovery. At the end of the pre-established contact time, each bag containing the ion exchanger was removed and the system was filtered. The obtained solution was subjected to ICP-MS analysis.

## 3. Results

### 3.1. Characterization of Zinc Ferrite

#### 3.1.1. ICP-MS: Elementary Analysis

The zinc ferrite was placed into solution, before being subjected to ICP-MS analysis, as described in Section 2.3. Table 2 shows the elements detected by the instrument. Analyses highlighted a variety of elements including heavy metals. The most abundant elements were: Fe, Zn, Pb, Ca, and Mn. The analyses confirmed the potential hazard of the waste: the percentages of Pb were are high.

#### 3.1.2. Scanning Electron Microscopy and Microanalysis

**Figure 1:**
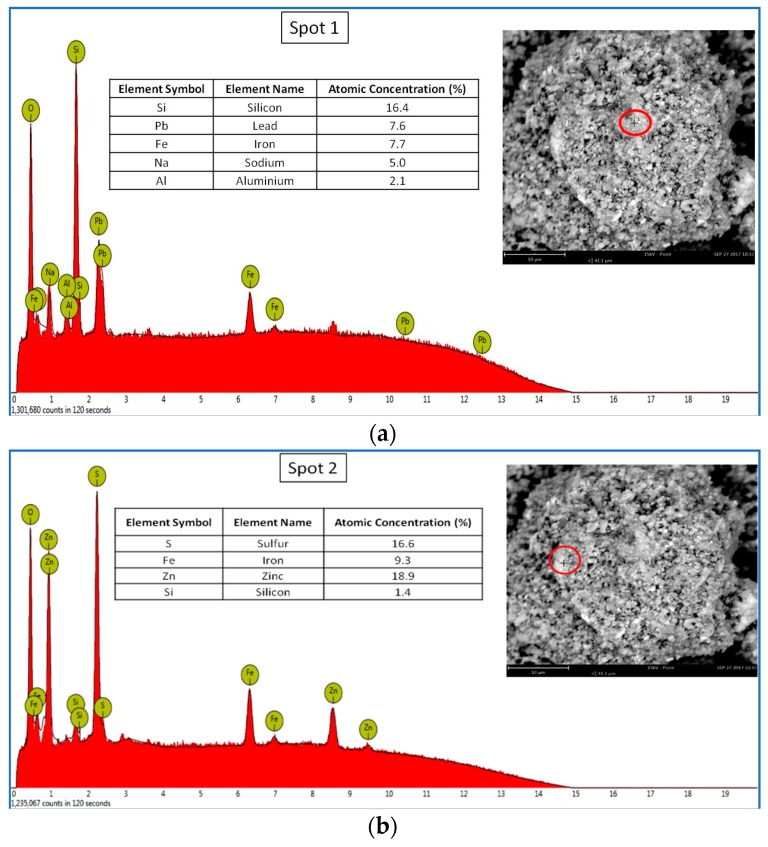

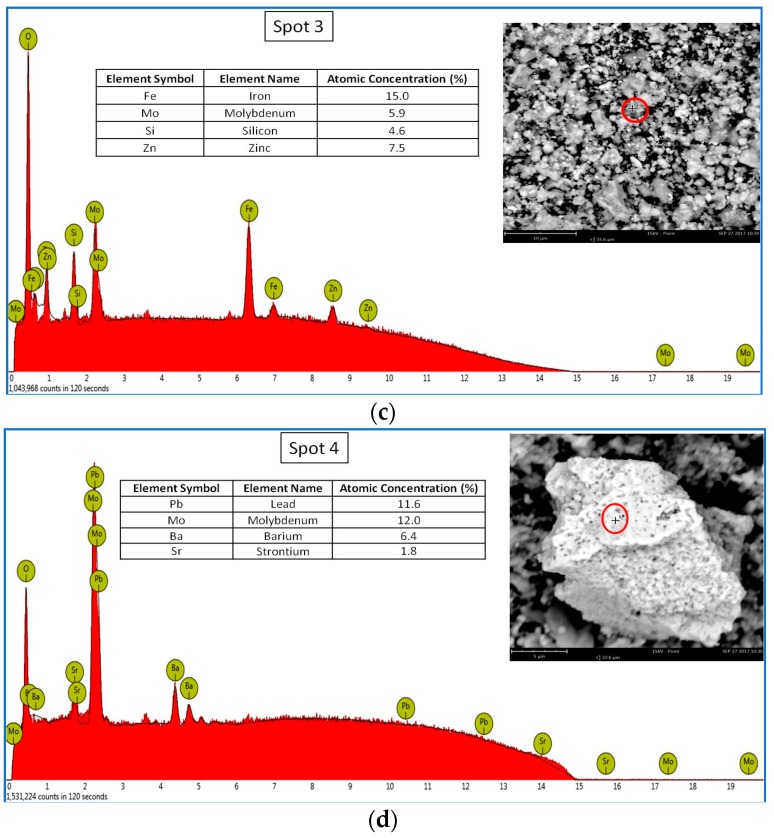
SEM images and EDS elementary analysis of zinc ferrite on different observation spots: (**a**) spot 1; (**b**) spot 2; (**c**) spot 3; (**d**) spot 4.

Figure 1 shows the SEM images and the EDS microanalysis of zinc ferrite sample (four spots). SEM images showed that the zinc ferrite, from a morphological point of view, is a heterogeneous material composed of many aggregates. Furthermore, the EDS analyses confirmed this heterogeneity from a chemical composition point of view. The different aggregates presented with a variable chemical composition and the presence of different elements. The most common elements were Fe, Zn, and Pb, which confirm their abundance due to the results obtained with ICP-MS analysis.

#### 3.1.3. X-ray Diffraction Analysis

**Figure 2:**
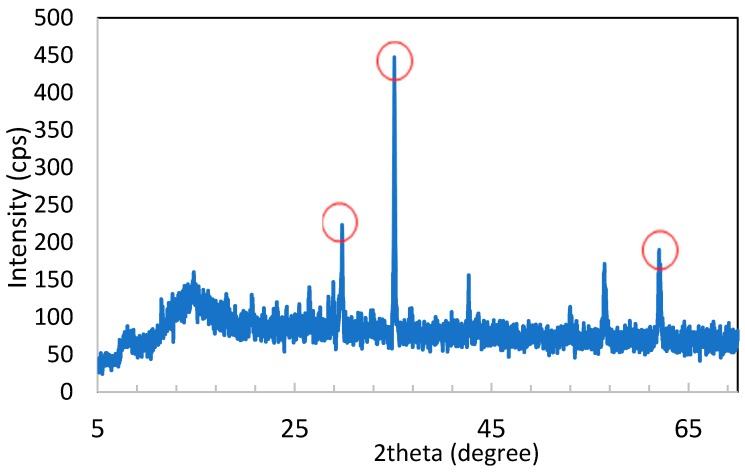
XRD spectrum of zinc ferrite and highlighted peaks attributable to the Franklinite phase.

XRD analysis was carried out on the zinc ferrite sample in order to identify the phases present in the solid (Figure 2). The observed XRD spectrum showed three intense characteristic peaks (identify also in the Table 3), which were attributed to the Franklinite phase (Zn, Mn, Fe) (Fe, Mn)_2_O_4_, in agreement with the nature of the elements present in zinc ferrite and measured by ICP-MS.

#### 3.1.4. Thermal Analysis

**Figure 3:**
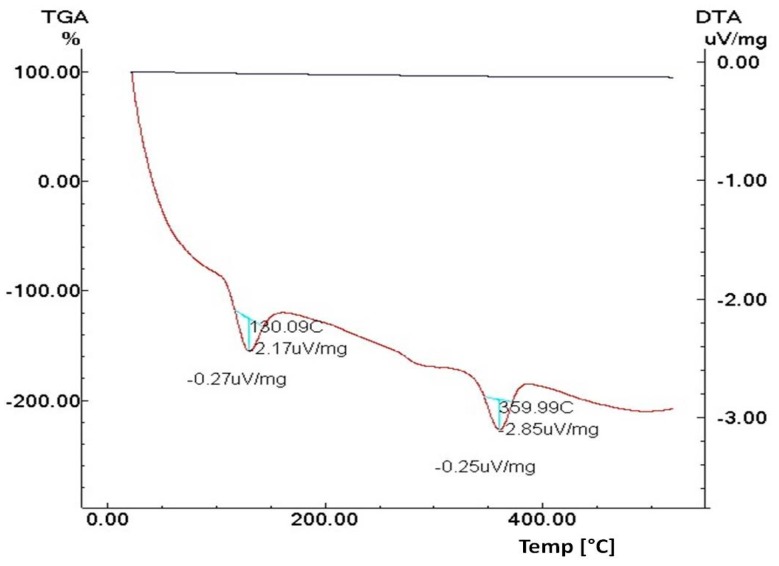
Thermal analysis, DTA (brown trace) and TGA (black trace), of zinc ferrite.

Figure 3 shows the TGA and DTA carried out on the zinc ferrite and conducted up to 500 °C in static air with a heating rate of 20 °C/min. The TGA confirmed, as expected, the lack of weight loss, whereas the two enthodermic peaks were attributale to phase transitions based on the DTA curve.

#### 3.1.5. pH and Conductivity

**Figure 4:**
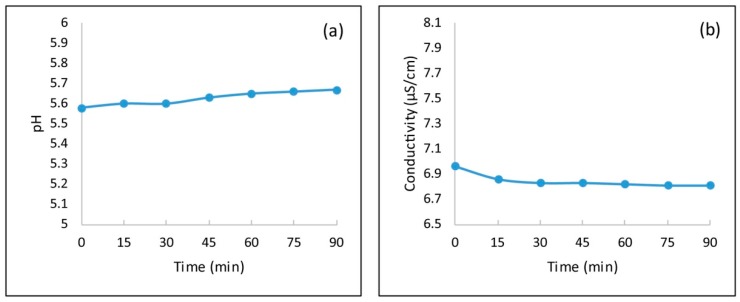
(**a**) pH and (**b**) conductivity of the zinc water-ferrite system as a function of the stirring time and at room temperature.

In order to study the behavior of zinc ferrite in water, pH and conductivity were measured in aqueous systems. The measurements were carried out at different times (Figure 4).

The obtained results showed that the zinc ferrite in water determines a pH of ca. 5.5, which remained constant. Furthermore, the conductivity was constant over a period of time with a value of ca. 6.8 μS/cm.

### 3.2. Removal of Elements Released by Zinc Ferrite in Aqueous Systems by ETS-10 Ion Exchanger

After having characterized the zinc ferrite, we started the next phase, which included the removal of the elements released by the zinc ferrite in water, using ETS-10 as the ion exchanger. Due to the complexity of the elements present in the zinc ferrite, which emerged from the elementary analysis shown in Table 2, we concentrated on studying the elements Fe, Mg, Ca, Zn, Mn, and Pb, which were the most abundant elements released into the water. The concentrations of Na and K were also tested, since they represent the cations that the ETS-10 phase exchanges. For this reason, we have prepared the blanks systems, containing only zinc ferrite in water (systems A—see Section 2.3). The concentrations of the elements in system A as a function of contact time, are shown in Table 4.

The data reported in Table 4 show that the concentrations of the elements released in water from the zinc ferrite were, in decreasing order: Zn > Ca > Mn > Na > Mg > K > Fe > Pb. The concentrations of these elements remained more or less constant over time. The concentrations, after 0.5 h, remained similar to those after 2 h of contact time. Most of the element concentrations were released quickly in a half hour, indicating the important releasing ability of zinc ferrite. The slight fluctuations in the concentrations, which were recorded over time, can be justified by the complexity of the system, given its many elements, and consequently the establishment of many equilibrium reactions.

In Table 5, the released percentages of each individual element are provided in terms of the quantity actually present in the zinc ferrite, taken at room temperature after two hours in water under constant stirring.

The above data show that the percentage of elements released into solution was not high compared to the percentages present in the zinc ferrite, confirming their low solubility. For example, in the case of Pb, only 5.47 × 10^−5^% was released into solution. This indicates a potential danger, because high quantities still remain, which could be released later on, or in other particular conditions. The elements that were released most abundantly in water from zinc ferrite were not those most abundant in it.

As far as iron and zinc are concerned, even though they are the most abundant elements in zinc ferrite, only 3.55 × 10^−5^% and 1.3 × 10^−2^% were released into solution, respectively.

The concentrations of the elements in systems B, as a function of contact time, are shown in Table 6. Data shows that even after 30 min, the presence of ETS-10 generally and substantially reduced the concentrations of the elements, except for sodium and potassium. This, as expected, was due to the ETS-10 phase, as it releases sodium and potassium during the exchange. After half hour, the concentration of the elements changed compared to the system without an ion exchanger (systems A), in the following decreasing order: Na > K > Ca > Mg > Zn > Fe > Pb.

### 3.3. Comparison between the ETS-10 Phase and Commercial Zeolite A (LTA) in the Removal of Metals from Water Contaminated by Zinc Ferrite

To compare the specific attitudes of the ETS-10 phase in the treatment of water contaminated by zinc ferrite with other materials, zeolite A was taken as a reference material and used in systems identical to those used in the presence of ETS-10 phase.

**Figure 5:**
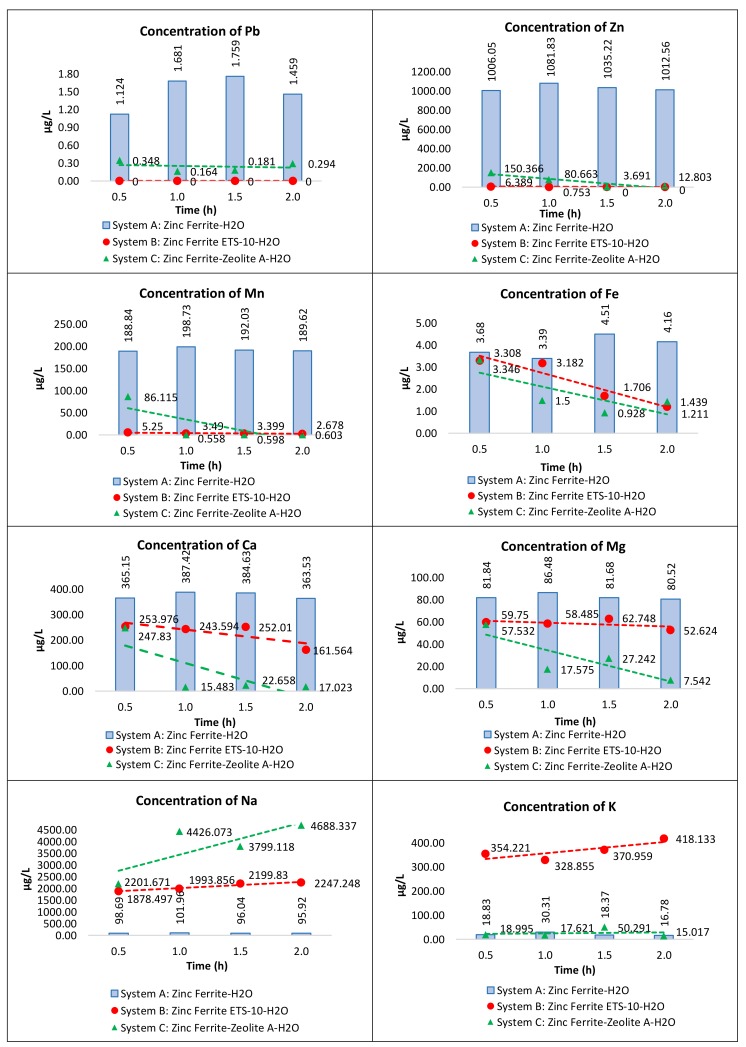
Comparison of the element concentration in System A (Zinc ferrite and water), System B (Zinc ferrite, ETS-10 and water) and System C (Zinc Ferrite, Zeolite A and water).

In order to appreciate the variation in the concentrations of the elements before and after treatment with ETS-10 and zeolite A, the concentrations of the elements in the three systems (blanks included) at different contact times were compared and reported for each individual element in Figure 5.

For both materials, the concentrations of Pb, Zn, Mn, Fe, Ca and Mg in the treated solution decreased as a function of contact time, whereas, in the case of sodium and potassium, the concentrations increased, confirming the exchange action.

By the ETS-10 ion exchanger, all Pb, Zn and Mn released were completely removed after only 0.5 h. While zeolite A is not able to completely reduce the Pb concentration, for the overall contact time analyzed, more than one hour to remove all Zn and Mn present is needed.

Regarding the removal of Fe, both materials showed almost similar behavior, but zeolite A resulted in a slightly more activity, especially at the beginning of treatment. The good ability of zeolite A towards Fe adsorption was already observed in a recent study [69].

Towards Ca and Mg, zeolite A always shows a better performance with respect to the ETS-10 material. This is not a surprise because it is well known that zeolite A is one of the best ion exchangers for water hardness reduction [72].

Table 7 shows the metals removal efficiency of ETS-10 and zeolite A for Pb, Zn, Mn, Fe, Ca and Mg after two hours of exchange time. We observed again, but in a more precise way, that the Pb, Zn, and Mn concentrations decreased by almost 100% compared to their initial concentrations, for systems treated with ETS-10.

On the contrary, zeolite A did not show efficiency over 70% for lead, 85% for zinc and 55% for manganese. After two hours of treatment, the efficiency of Zeolite A and EST-10 towards Fe removal was practically the same, ca. the 11%. While in the removal of calcium and magnesium, the best performance of zeolite A is confirmed also after longer treatment times.

The different selectivity of ETS-10 material towards metals removing could be explained considering the real size of cations. Particularly, symmetry and hydrated radius of the cations in the solution are important parameters to consider. In Table 8, we have reported hydrated radii of the metals removed [73] and the corresponding selectivity of the ETS-10 ion exchanger. 

It is possible to observe that, at the increasing of the hydrated radius of the ions, the corresponding metal removal efficiency of ETS-10 decreases. This aspect can explain why the Pb is completely and quickly removed by water with respect to the Zn, Mn and Fe; the dimension of these last ions in their hydrated state is larger than that of the Pb. Moreover, a recent study demonstrated that the structure of hydrated lead(II) ions, with respect to zinc(II), manganese(II), iron(II) and iron(III) ions, possess a lower symmetry (hemi-directed) [74]. So, the Pb removal is favored with respect to that of Zn, Mn and Fe.

The influence of steric hindrance of hydrated ions is much more evident for ETS-10 material with respect to zeolite A because their different pores size dimension. Since the pore size of zeolite A is close to 3 Å, all metals are removed only by its external surface. While ETS-10, with a pore dimension higher than that of zeolite A and close to ca. 8 Å [26,27], is able to remove bigger hydrated ions using also its internal surface. This favors the complete removal of ions with hydrated radii comparable to its pores size and, of course, the ion exchange of cations with lower hydrated radius is faster.

Notably, the ETS-10 after use can be easily obtained again in the sodium and potassium initial form by a subsequently ion exchange. The ETS-10 phase does not change after the treatment and is not affected by the use and regeneration processes [22,42].

If we consider the following formula (Na_1.5_K_0.5_)TiSi_5_O_13_ of the anhydrous ETS-10 phase [24] and the total moles of elements released by the zinc ferrite in water after one hour, i.e., Zn^2+^, Ca^2+^, Mn^2+^, Mg^2+^, Fe^2+^, and Pb^2+^ (Table 4), excluding Na^+^ and K^+^ because ions released from the ETS-10 phase during the exchange, it is possible to estimate the theoretical quantity of ETS-10 necessary to purify one cubic meter of water. This theoretical quantity is ca. 15 g of ETS-10 per cubic meter of contaminated water.

Therefore, considering the small amount of ETS-10 necessary for the purification process, its simple regeneration, and the low cost of raw materials necessary for its preparation, the overall purification process can be considered economical. However, a precise value of the costs can be provided only after an appropriate feasibility study of the process, but this was not the purpose of the present work.

The efficiency of the ion exchangers depends on many parameters, such as pH, metal concentration, and type of pretreatment, and therefore, on the general characteristics of the system in which the exchanger operates [20]. Zinc ferrite/water is a complex system. Many substances and elements are present in this system that can produce many balanced reactions which can modify the effectiveness of the ion exchanger. Demonstrating that ETS-10 is an efficient material for zinc ferrite/water purification was the goal of this work, especially considering that its efficiency is higher than that produced by common exchangers, like zeolite A in the same and complex water system.

## 4. Conclusions

From the obtained results, we concluded that the characterization of zinc ferrite by ICP-MS showed that it is a material rich in many elements. The most abundant were iron, zinc, lead, calcium, aluminum and manganese. The XRD analysis showed more intense characteristic peaks attributable to the Franklinite phase. Using scanning electron microscopy and microanalysis, it was observed that the material is heterogeneous in regards to the morphology and chemical composition of the aggregates present.

Zinc ferrite in water and at room temperature, induced a slightly acidic pH, around 6, and released some elements including Zn, Ca, Mn, Na, Mg, K, Fe, and Pb. The percentage of the elements released in water as opposed to their quantity actually present in the zinc ferrites confirms the low solubility of zinc ferrite as a whole. However, this last aspect makes this material potentially more dangerous because there are, indeed, higher quantities that could be released with time or under more severe conditions and in a constant manner.

The ETS-10 demonstrated its high efficiency for metal removal. All metals present in the water were significantly removed by ETS-10. If it is compared with commercial zeolite A, ETS-10 shows a better efficiency for the removal of lead, zinc and manganese, in fact, after only about 30 min there is a reduction close to 100%. Iron, calcium and manganese removal efficiencies of ETA-10 and zeolite A were comparable.

The metal removal efficiency is affected by the symmetry and hydrated radius of the ions in solution. Among all metals present in the wastewater, lead(II) possesses the lowest hydrated radius and, hence, is quickly and completely removed by ETS-10 ion exchanger.

The increase in Na^+^ and K^+^ concentrations confirms the exchange between zinc ferrite and ETS-10. The ion exchanger ETS-10, never previously tested in this type of treatment, was efficient for the treatment of water contaminated by zinc ferrite. The material preserves its functionality and its integrity in a complex system, zinc ferrite/water, in which there are many elements, substances, and equilibrium reactions.

The ETS-10 material is potentially competitive with other similar materials in this type of treatment because it possesses all the characteristics required of an ion exchanger; high efficiency, stability, regeneration, and economy. Since there is currently no real market for ETS-10 material, further Life Cycle Assessment studies of ETS-10 production and application in water treatment are warranted.

## Figures and Tables

**Table 1:** Scheme of prepared systems for metal removal.

Systems	Time (h)	System Composition
1A	0.5	5 g Zinc ferrite + 100 mL H_2_O
2A	1.0	
3A	1.5	
4A	2.0	
1B	0.5	5 g Zinc ferrite + 10 g ETS-10 + 100 mL H_2_O
2B	1.0	
3B	1.5	
4B	2.0	
1C	0.5	5 g Zinc ferrite + 10 g zeolite A + 100 mL H_2_O
2C	1.0	
3C	1.5	
4C	2.0	

**Table 2:** ICP-MS data: Concentrations of the elements of a solution containing 0.5 g zinc ferrite (Section 2.3).

Element	Concentration (mg/L)	% Weight
Fe	1168.000	23.360
Zn	768.000	15.360
Pb	265.000	5.300
Ca	103.000	2.060
Mn	59.800	1.196
Al	31.300	0.626
Mg	19.000	0.380
Cu	15.300	0.306
Na	14.800	0.296
Sr	11.100	0.222
K	8.000	0.160
Cd	3.070	0.061
Ba	1.950	0.039
In	1.130	0.026
Ni	0.559	0.011
Cr	0.549	0.011
Ga	0.450	0.009
Co	0.302	0.006
Li	0.020	4 × 10^−4^
Rb	0.018	3.6 × 10^−4^
Cs	0.009	1.8 × 10^−4^

**Table 3:** Characteristic peaks of zinc ferrite and attributed to Franklinite.

Peak	2theta (degree)	Intensity (cps)	d-Spacing
1	29.8	227.1429	2.5546
2	35.1	440.1429	2.9957
3	61.98	190.3463	1.4961

**Table 4:** Concentrations (µg/L) of different elements in systems A.

	Concentration (µg/L)			
Time	0.5 h	1.0 h	1.5 h	2.0 h
Zn	1006.045	1081.831	1035.219	1012.561
Ca	365.153	387.416	384.632	363.531
Mn	188.840	198.733	192.026	189.617
Na	98.691	101.962	96.040	95.917
Mg	81.840	86.480	81.676	80.522
K	18.829	30.309	18.367	16.780
Fe	3.683	3.387	4.505	4.158
Pb	1.124	1.681	1.759	1.459

**Table 5:** Percentage of release of elements after two hours in water, with constant stirring, in terms to their quantity present in zinc ferrite.

Element	Wt % Released
Na	6.48 × 10^–2^
Mg	4.24 × 10^–2^
Ca	3.26 × 10^–2^
Mn	3.17 × 10^–2^
K	2.09 × 10^–2^
Zn	1.32 × 10^–2^
Pb	5.47 × 10^–5^
Fe	3.55 × 10^–5^

**Table 6:** Concentrations (µg/L) of different elements in System B containing zinc ferrite, ETS-10 and water at different times.

	Concentration (µg/L)			
Time	0.5 h	1.0 h	1.5 h	2.0 h
Na	1878.497	1993.856	2199.830	2247.248
K	354.221	328.855	370.959	418.133
Ca	253.976	243.594	252.010	161.564
Mg	59.750	58.485	62.748	52.624
Zn	6.389	0.753	0.000	0.000
Mn	5.250	3.490	3.399	2.678
Fe	3.308	3.182	1.706	1.211
Pb	0.000	0.000	0.000	0.000

**Table 7:** Metals removal efficiency of ETS-10 phase (System B) and of the zeolite A (System C) after half hour.

Element	Efficiency	
	ETS-10	Zeolite A
Pb	100.00%	69.10%
Zn	97.71%	84.95%
Mn	97.65%	54.38%
Fe	10.45%	10.70%
Ca	30.22%	32.41%
Mg	26.98%	29.61%

**Table 8:** Hydrated radius of the ions and corresponding metals removal efficiency of ETS-10 phase.

Element	Efficiency	
	ETS-10	Hydrated Radii (Å) [73]
Pb^2+^	100.00%	4.01
Zn^2+^	97.71%	4.30
Mn^2+^	97.65%	4.38
Fe^2+^/Fe^3+^	10.45%	4.28/4.57
